# Idiopathic Multicentric Castleman Disease Mimicking Sepsis and Autoimmune Disease in a Caribbean Patient

**DOI:** 10.7759/cureus.117229

**Published:** 2026-09-30

**Authors:** Amber S Maharaj

**Affiliations:** 1 Internal Medicine, Eric Williams Medical Sciences Complex, Ministry of Health, St. Augustine, TTO

**Keywords:** autoimmune like, idiopathic multicentric castleman disease, inflammatory cytokines, multicentric castleman disease, orphan diseases

## Abstract

Idiopathic multicentric Castleman disease (iMCD) is a rare cytokine-driven lymphoproliferative disorder characterised by systemic inflammation, generalised lymphadenopathy, and multiorgan involvement. Its clinical presentation may closely mimic sepsis, autoimmune disease, and other inflammatory disorders, creating a significant diagnostic challenge, particularly in resource-limited settings. We describe a 73-year-old Caribbean woman who presented with approximately one month of fever, progressive unintentional weight loss, profound fatigue, bilateral lower-limb oedema, and cutaneous hyperpigmentation. She was initially evaluated for sepsis and treated with broad-spectrum antibiotics for one week; however, microbiological investigations were negative, and there was no significant clinical improvement. Laboratory investigations demonstrated marked systemic inflammation, anaemia, thrombocytopenia, leukopenia, and hypoalbuminaemia. Computed tomography revealed generalised lymphadenopathy and splenomegaly, with additional thoracic findings including interstitial lung changes and small pleural and pericardial effusions. Autoimmune serology showed isolated ANA positivity, with dsDNA, ENA subtypes, rheumatoid factor, anti-CCP, and ANCA all negative - not meeting criteria for an alternative connective tissue disease. Excisional lymph node biopsy demonstrated atrophic germinal centres, expanded mantle zones, and polyclonal plasmacytosis, while testing for human herpesvirus-8 and immunoglobulin G4 was negative, confirming iMCD. A concurrent breast mass identified on imaging was biopsied and showed features in keeping with iMCD rather than malignancy. Treatment included corticosteroids, hydroxychloroquine, and azathioprine, with supportive therapy. During a severe inflammatory flare six weeks after admission, tocilizumab produced transient clinical and biochemical improvement, but she developed pruritus, angioedema, and shortness of breath during infusion; thus, ongoing biologic therapy was not continued. She has since been followed for approximately 19 months with a relapsing course managed through sequential steroid-sparing agents, with improved fatigue, weight gain, and improved quality of life despite residual cutaneous changes. This case highlights the importance of considering iMCD in patients with persistent systemic inflammation, generalised lymphadenopathy, cytopenias, and a negative infectious work-up and non-lupus-specific autoimmune profile, and illustrates the practical challenges of sustaining guideline-directed biologic therapy in a resource-limited Caribbean setting.

## Introduction

Castleman disease (CD) comprises a group of rare lymphoproliferative disorders characterised by distinctive lymph node histopathology and heterogeneous clinical manifestations. iMCD is estimated to affect fewer than one to two people per million per year, most commonly presenting in the fifth to seventh decades of life, though it can occur at any age. The disease is broadly classified into unicentric CD and multicentric CD, with multicentric disease further categorised into human herpesvirus-8 (HHV-8)-associated disease, HHV-8-negative idiopathic multicentric Castleman disease (iMCD), and Polyneuropathy, Organomegaly, Endocrinopathy, Monoclonal gammopathy, and Skin changes (POEMS)-associated multicentric CD, a distinct paraneoplastic syndrome driven by an underlying plasma-cell clone rather than the cytokine dysregulation seen in iMCD [1,2].

iMCD is a rare and potentially life-threatening inflammatory disorder characterised by generalised lymphadenopathy, systemic inflammation, polyclonal lymphoproliferation, and variable multiorgan dysfunction [1-3]. The pathophysiology of iMCD involves dysregulated immune and cytokine signalling, with interleukin-6 (IL-6) playing an important role in the systemic inflammatory manifestations of the disease [1,3]. Clinical features may include fever, fatigue, weight loss, anaemia, thrombocytopenia, elevated inflammatory markers, hypoalbuminaemia, oedema, hepatosplenomegaly, pulmonary abnormalities, and serosal effusions [1,4]. Because these manifestations are non-specific, iMCD may closely resemble infection, malignancy, systemic autoimmune disease, or other inflammatory disorders [3-5].

The diagnosis of iMCD requires integration of clinical, radiological, laboratory, and histopathological findings. International consensus diagnostic criteria require both major criteria - multicentric lymphadenopathy and characteristic lymph node histopathology - together with at least two minor criteria, including at least one laboratory abnormality, and exclusion of relevant infectious, malignant, and autoimmune mimics [5]. This diagnostic framework is particularly important in rheumatology, where iMCD may present with autoimmune-like manifestations or coexist with autoimmune disease [6].

The clinical overlap between iMCD and systemic autoimmune disease is particularly important in rheumatological practice. Patients may demonstrate constitutional symptoms, cytopenias, lymphadenopathy, skin manifestations, elevated inflammatory markers, and organ involvement that can resemble systemic lupus erythematosus (SLE) and other connective tissue diseases [6]. Recent reports of patients with SLE and CD further demonstrate the diagnostic complexity created by this clinical overlap [7,8].

This case describes a 73-year-old Caribbean woman whose presentation initially raised concern for sepsis and subsequently demonstrated significant overlap with systemic autoimmune disease. The case illustrates the diagnostic difficulty associated with persistent systemic inflammation, cytopenias, generalised lymphadenopathy, and multisystem involvement, and highlights the practical challenges of implementing guideline-directed biologic therapy in a Caribbean healthcare setting.

This case report was previously presented as a poster at the Society for Acute Medicine (SAMCardiff) annual conference in May 2026.

## Case presentation

A 73-year-old Caribbean woman presented with a one-month history of fever, progressive unintentional weight loss, profound fatigue, marked bilateral lower-limb oedema, and cutaneous hyperpigmentation. She also experienced sensory symptoms involving the peripheral limbs. Her presentation was concerning for a significant systemic inflammatory process, and sepsis was initially considered among the differential diagnoses.

On admission, she was hypotensive and had evidence of anaemia, thrombocytopenia, markedly elevated inflammatory markers, and hypoalbuminaemia. A baseline sepsis screen was performed, including blood and urine cultures, respiratory sampling, and a septic-focus examination, alongside routine haematology, biochemistry, and autoimmune serology. Broad-spectrum antibiotic therapy was initiated empirically for presumed infection and continued for one week. Subsequent microbiological investigations demonstrated no evidence of active infection, and there was no significant clinical improvement with antimicrobial therapy, prompting reconsideration of the working diagnosis.

Laboratory investigations demonstrated persistent systemic inflammation, anaemia, thrombocytopenia, leukopenia, and hypoalbuminaemia. Haemoglobin ranged from 8.9 to 10.2 g/dL (UK reference range, 11.5-16.5 g/dL), white cell count ranged from 2.8 to 3.6 × 10⁹/L (reference range, 3.6-11.0 × 10⁹/L), and platelet count ranged from 90 to 130 × 10⁹/L (reference range, 140-400 × 10⁹/L). The erythrocyte sedimentation rate remained greater than 120 mm/h (reference range, 5-15 mm/h), while C-reactive protein ranged from 85 to 110 mg/L (reference range, <5 mg/L). Serum albumin was reduced (reference range, 35-50 g/L). Complement levels were reduced, with C3 below the reference range of 0.90-1.80 g/L and C4 below the reference range of 0.10-0.40 g/L. Renal function was preserved, with creatinine and eGFR within normal limits throughout. Autoimmune serology comprised ANA, anti-dsDNA, ENA subtypes, rheumatoid factor, anti-CCP, and ANCA; only ANA was positive, with all other autoantibodies negative. This isolated, non-lupus-specific ANA positivity did not meet criteria for an alternative connective tissue disease.

Computed tomography demonstrated generalised lymphadenopathy and splenomegaly. Thoracic imaging additionally demonstrated interstitial lung changes with small bilateral pleural effusions and a minor pericardial effusion. The patient had prominent cutaneous manifestations, including chronic hyperpigmented vascularised plaques and indurated inflammatory skin changes. Peripheral oedema, sensory neuropathy, and recurrent periorbital cellulitis were also documented during disease flares. As part of the malignancy work-up prompted by her age and constitutional symptoms, a mass identified on high-resolution CT of the chest was biopsied; histopathology showed features in keeping with iMCD (Castleman-type lymphoid tissue exhibiting hyperplastic germinal centres, hypervascularity and plasmacytosis) rather than a primary breast malignancy, excluding malignancy at that site. Pulmonary function testing, echocardiography, and electrocardiography were also performed as part of the broader systemic work-up and did not raise concern for an occult malignancy.

Figure 1 illustrates the computed tomography findings. 

**Figure 1:**
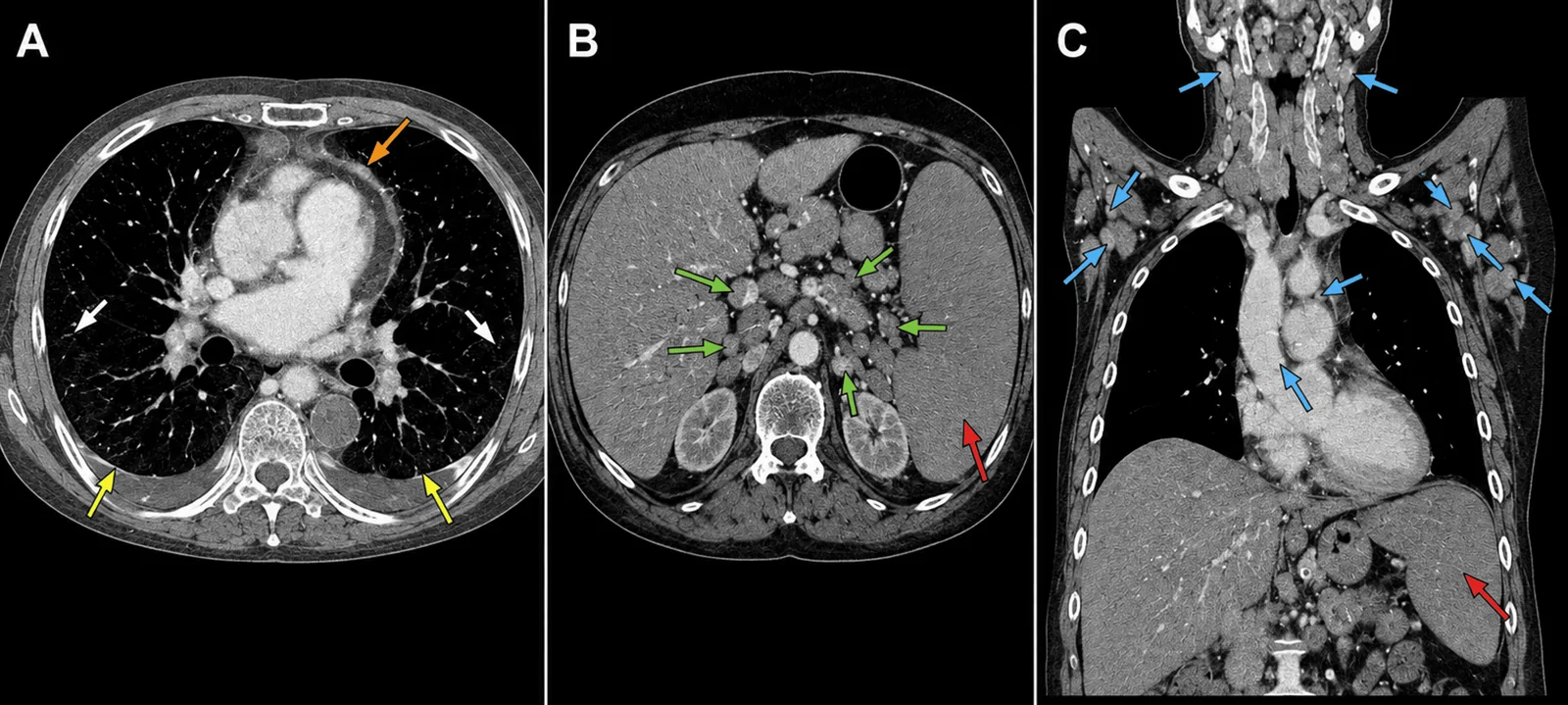
Computed tomography (CT) demonstrating generalised lymphadenopathy and splenomegaly with associated multiorgan manifestations of idiopathic multicentric Castleman disease (iMCD) (A) Axial contrast-enhanced CT of the chest demonstrating bilateral interstitial lung changes, small bilateral pleural effusions, and a small pericardial effusion, with the orange arrow indicating the pericardial effusion and the white and yellow arrows indicating the bilateral pleural effusions and interstitial lung changes, respectively. (B) Axial contrast-enhanced CT of the abdomen demonstrating splenomegaly and multiple enlarged retroperitoneal and mesenteric lymph nodes, with the green arrows indicating enlarged retroperitoneal and mesenteric lymph nodes and the red arrow indicating the enlarged spleen. (C) Coronal contrast-enhanced CT of the chest and upper abdomen demonstrating generalised lymphadenopathy involving the cervical, supraclavicular, mediastinal, and axillary regions, with splenomegaly, with the blue arrows indicating generalised lymphadenopathy across the cervical, supraclavicular, mediastinal, and axillary regions and the red arrow again indicating splenomegaly.

Given the persistent systemic inflammation, generalised lymphadenopathy, cytopenias, a non-lupus-specific autoimmune profile, and absence of microbiological evidence of active infection, haematology and rheumatology assessment was pursued. An excisional lymph node biopsy demonstrated atrophic germinal centres, expanded mantle zones, and polyclonal plasmacytosis. On overall review, the nodal architecture, vascular changes, and plasma-cell population were considered in keeping with iMCD, with no features to suggest an alternative diagnosis. 

HHV-8 and IgG4 testing were negative. The combination of multicentric lymphadenopathy, characteristic lymph node histopathology, supportive clinical and laboratory features, and exclusion of alternative diagnoses established the diagnosis of iMCD [5], as summarised against the international consensus diagnostic criteria in Table 1, with the differential diagnosis and distinguishing features set out in Table 2.

**Table 1:** Fulfilment of the 2017 international consensus diagnostic criteria for HHV-8-negative/idiopathic multicentric Castleman disease eGFR, estimated glomerular filtration rate; HHV-8, human herpesvirus 8; SPEP, serum protein electrophoresis

Criterion	Type	Present in this patient
Multicentric lymphadenopathy (≥2 stations)	Major	Yes - cervical, supraclavicular, mediastinal, axillary, retroperitoneal, mesenteric nodes on CT
Characteristic lymph node histopathology	Major	Yes - atrophic germinal centres, expanded mantle zones, polyclonal plasmacytosis; HHV-8 and IgG4 negative
Elevated CRP/ESR	Minor (lab)	Yes - CRP 85-110 mg/L; ESR >120 mm/h
Anaemia	Minor (lab)	Yes - Hb 8.9-10.2 g/dL
Thrombocytopenia or thrombocytosis	Minor (lab)	Yes - platelets 90-130 × 10⁹/L
Hypoalbuminaemia	Minor (lab)	Yes
Renal dysfunction/eGFR reduction	Minor (lab)	No - renal function preserved (creatinine/eGFR within normal limits)
Polyclonal hypergammaglobulinaemia	Minor (lab)	Not performed (SPEP/globulin not obtained in our setting)
Constitutional symptoms (fever, night sweats, weight loss, fatigue)	Minor (clinical)	Yes - fever, weight loss, fatigue
Hepatomegaly and/or splenomegaly	Minor (clinical)	Yes - splenomegaly
Fluid accumulation (oedema, ascites, pleural/pericardial effusion)	Minor (clinical)	Yes - peripheral oedema, pleural and pericardial effusions
Skin changes (cherry haemangiomas, violaceous papules, etc.)	Minor (clinical)	Yes - hyperpigmented vascularised plaques, indurated inflammatory skin changes

**Table 2:** Differential diagnoses and distinguishing features HHV-8, human herpesvirus 8; iMCD, idiopathic multicentric Castleman disease; POEMS, Polyneuropathy, Organomegaly, Endocrinopathy, Monoclonal gammopathy, and Skin changes; SPEP, serum protein electrophoresis

Differential diagnosis	Typical findings	Findings in this patient
Sepsis/severe infection	Positive cultures or an identifiable focus; clinical response to appropriate antimicrobial therapy	Blood, urine, and respiratory cultures negative; no clinical improvement after one week of broad-spectrum antibiotics
Systemic lupus erythematosus/other connective tissue disease	Lupus-specific autoantibodies (anti-dsDNA, anti-Smith, other ENA subtypes), often with anti-Ro/La or RF/anti-CCP positivity	Only ANA positive; anti-dsDNA, ENA subtypes, RF, anti-CCP, and ANCA all negative
HHV-8-associated multicentric Castleman disease	Positive HHV-8 testing on lymph node tissue, often in the context of HIV infection	HHV-8 testing negative
IgG4-related disease	Positive IgG4 immunostaining/serology; storiform fibrosis and obliterative phlebitis on histology	IgG4 testing negative; histology showed atrophic germinal centres, expanded mantle zones, and polyclonal plasmacytosis rather than IgG4-RD features
POEMS-associated multicentric Castleman disease	Monoclonal gammopathy on SPEP, polyneuropathy, endocrinopathy, and characteristic skin changes	Clinical picture did not otherwise suggest POEMS; SPEP was not performed in our setting, a limitation noted in Table 1
Lymphoma/other malignancy	Monomorphic lymphoid infiltrate or malignant cells on tissue biopsy	Excisional lymph node biopsy showed reactive, polyclonal plasmacytic changes rather than a lymphomatous infiltrate; a separate breast mass biopsy showed iMCD-type change rather than malignancy

Initial treatment consisted of prednisolone 40 mg daily followed by gradual tapering, with hydroxychloroquine 200 mg twice daily. Azathioprine up to 100 mg daily was introduced as steroid-sparing therapy after approximately three months of corticosteroid therapy and tapering. Gabapentin 300 mg three times daily was prescribed for neuropathic symptoms. During a severe inflammatory flare occurring approximately six weeks after her initial admission, intravenous tocilizumab at 8 mg/kg was administered and resulted in transient clinical and biochemical improvement. During the infusion, she developed pruritus, angioedema, and shortness of breath, consistent with an acute hypersensitivity/infusion reaction; the infusion was discontinued, and she was managed supportively per standard infusion-reaction protocol. Siltuximab, the preferred first-line anti-IL-6 agent internationally, was considered as an alternative but was not accessible in our healthcare setting. Continued biologic therapy was therefore not pursued.

Longitudinal follow-up

The patient has since been followed in rheumatology clinic over approximately 19 months. Her course has been relapsing, marked by recurrent lymphadenopathy that has settled intermittently, persistently low C3/C4, and fluctuating cytopenias (haemoglobin as low as 9.1 g/dL, white cell count as low as 2.8-3.6 × 10⁹/L, platelets as low as 90-200 × 10⁹/L, ESR persistently >120 mm/h on repeated testing). She has required sequential steroid-sparing agents, moving from azathioprine to mycophenolate mofetil (up to 500 mg twice daily) during subsequent flares, alongside an oral corticosteroid taper. She has had recurrent respiratory and sinus infections, managed with courses of oral antibiotics (e.g., cefuroxime). Suspected mononeuritis multiplex and cutaneous vasculopathic change/panniculitis have been identified on subsequent skin biopsy during follow-up, kept under joint rheumatology surveillance alongside the iMCD diagnosis. Following her infusion reaction, she declined further tocilizumab; rituximab remains under consideration as a further treatment option pending response to current therapy, therapies which may be unavailable in a resource-limited Caribbean setting. 

## Discussion

iMCD presents a substantial diagnostic challenge because of its rarity, heterogeneous clinical manifestations, and overlap with infectious, autoimmune, malignant, and other inflammatory disorders [1,3,5]. The 2024 case report by Schmalzing et al. similarly demonstrated how constitutional symptoms, cytopenias, systemic inflammation, and organ involvement can contribute to substantial diagnostic uncertainty [9].

In the present case, the initial presentation closely resembled severe infection, with fever, hypotension, inflammatory marker elevation, cytopenias, oedema, and multisystem involvement. Broad-spectrum antimicrobial therapy was therefore initiated while infection was investigated. However, microbiological investigations did not demonstrate active infection, and there was no significant clinical improvement. Persistent inflammation despite a week-long antimicrobial treatment prompted reconsideration of the initial diagnosis.

The clinical overlap with autoimmune disease was also considerable. The patient demonstrated constitutional symptoms, cytopenias, cutaneous manifestations, peripheral neurological symptoms, hypocomplementaemia, serosal effusions, and persistent systemic inflammation. These findings can raise concern for SLE or another connective tissue disease [6]. Practical guidance on iMCD and autoimmune conditions emphasises that autoimmune and autoinflammatory diseases may mimic iMCD and should be carefully excluded before the diagnosis is established [6].

Recent reports further illustrate this diagnostic overlap. Bravo-Andrade et al. described two cases of SLE associated with CD, demonstrating the diagnostic complexity created by overlapping autoimmune and lymphoproliferative manifestations [7]. Similarly, Ma et al. reported SLE combined with CD and secondary paraneoplastic pemphigus [8]. In this patient, autoimmune serology did not establish an alternative connective tissue disease at the time of the index diagnosis; her subsequent follow-up course, with suspected mononeuritis multiplex and features raising the question of an overlapping connective tissue process, illustrates that this diagnostic overlap can continue to evolve over time rather than being resolved at a single time point.

Histopathological diagnosis

Lymph node biopsy was pivotal in establishing the diagnosis. Characteristic histopathological abnormalities in iMCD may include regressed or abnormal germinal centres, mantle-zone changes, vascular proliferation, and polytypic plasmacytosis [5]. The diagnosis requires integration of these findings with multicentric lymphadenopathy and supportive clinical and laboratory features while excluding relevant mimics [5].

The negative HHV-8 testing was important in distinguishing idiopathic multicentric disease from HHV-8-associated multicentric CD. Similarly, negative IgG4 testing helped exclude IgG4-related disease as an alternative explanation for the lymphadenopathy and systemic inflammatory presentation.

The patient demonstrated several recognised minor clinical and laboratory features of iMCD, including elevated inflammatory markers, cytopenias, hypoalbuminaemia, oedema, constitutional symptoms, generalised lymphadenopathy, splenomegaly, pulmonary abnormalities, and serosal effusions [3-5], as summarised in Table 1.

Multisystem and cutaneous manifestations

The patient's multisystem manifestations demonstrate the broad clinical spectrum of iMCD. In addition to generalised lymphadenopathy and splenomegaly, she demonstrated cutaneous inflammatory changes, peripheral oedema, sensory neuropathic symptoms, interstitial lung changes, pleural effusions, and a pericardial effusion.

Cutaneous manifestations may contribute to diagnostic confusion because they can resemble vasculitic or connective tissue disease-associated skin disease. The presence of inflammatory and hyperpigmented lesions in this patient therefore contributed to the autoimmune differential and was echoed on subsequent follow-up when a further skin biopsy showed vasculopathic changes and panniculitis.

The combination of cutaneous manifestations, systemic inflammation, lymphadenopathy, and cytopenias should prompt consideration of systemic inflammatory and lymphoproliferative disorders when conventional autoimmune investigations are unrevealing.

Relevance to rheumatology practice

This case is particularly relevant to rheumatology because iMCD can closely resemble systemic autoimmune disease. CD may occur in association with SLE, while autoimmune diseases can also produce clinical and histopathological findings that resemble CD [6-8].

The rheumatological literature emphasises that early consideration of CD and close collaboration between clinicians and pathologists are important when patients present with unexplained systemic inflammation and lymphadenopathy [10]. In the present case, the diagnosis depended on maintaining a broad differential diagnosis and obtaining an excisional lymph node biopsy.

Management and the Caribbean healthcare context

Management of iMCD has evolved substantially with recognition of the central role of IL-6 signalling. International consensus treatment guidelines recommend anti-IL-6 therapy as the preferred first-line treatment for iMCD, with siltuximab recommended when available and tocilizumab considered when siltuximab is unavailable [11]. The efficacy of siltuximab has been demonstrated in a randomised controlled trial involving patients with symptomatic multicentric CD [12]. Tocilizumab has also demonstrated clinical benefit in multicentric CD [13].

In this case, tocilizumab was administered during a severe inflammatory flare and produced transient clinical and biochemical improvement. However, she developed an acute hypersensitivity/infusion reaction with pruritus, angioedema, and shortness of breath, and continued biologic therapy was not feasible; siltuximab, the preferred alternative, was not accessible in our setting. IL-6 levels themselves were not measured during her admission, as this assay was not available in our healthcare setting - a further illustration of the resource constraints discussed below. Management consequently required corticosteroids, sequential steroid-sparing immunosuppressive therapy, symptomatic treatment, and multidisciplinary follow-up.

The Caribbean setting adds an important dimension to this case. International treatment recommendations provide an evidence-based framework, but implementation can differ substantially between healthcare systems. In the United Kingdom, NHS England has established siltuximab as a treatment option for eligible adults with iMCD [12]. In contrast, this case demonstrates the practical challenges associated with sustained access to targeted biologic treatment in a Caribbean setting.

This difference should not be interpreted as a difference in clinical expertise. Rather, it illustrates how healthcare system resources, access to specialist therapies, treatment tolerability, and availability of specialised investigations can influence the translation of international guidelines into individual patient care.

Diagnostic and therapeutic implications

Several clinical lessons emerge from this case. First, persistent systemic inflammation should not automatically be attributed to infection when microbiological investigations are unrevealing, and there is inadequate response to antimicrobial therapy.

Second, iMCD should remain in the differential diagnosis of patients with generalised lymphadenopathy, cytopenias, constitutional symptoms, and multiorgan involvement, particularly when autoimmune investigations do not establish an alternative diagnosis [5,6].

Third, tissue diagnosis is central. Characteristic lymph node histopathology, interpreted alongside multicentric lymphadenopathy, supportive clinical and laboratory features, and exclusion of mimics, provides the foundation for diagnosis [5].

Early multidisciplinary involvement of internal medicine, rheumatology, haematology, pathology, radiology, and other relevant specialities may facilitate earlier diagnosis and appropriate management. In a rare disease, such as iMCD, recognition of the clinical pattern and timely tissue diagnosis may be as important as access to advanced therapeutic options.

Strengths and limitations

This case adds a detailed, longitudinally followed account of iMCD from a Caribbean healthcare setting, an under-represented context in the existing literature, and illustrates how resource availability shapes real-world implementation of international treatment guidelines. Its limitations include those inherent to a single-patient case report: findings cannot be generalised, some treatment responses are reported descriptively rather than with quantified pre/post biomarker values, IL-6 levels were not available for measurement, and several histopathological and temporal details were not fully captured in the file records available to the author at the time of writing.

## Conclusions

iMCD should be considered in patients presenting with persistent systemic inflammation, generalised lymphadenopathy, cytopenias, constitutional symptoms, and multisystem involvement, particularly when infectious investigations are negative and autoimmune testing does not establish an alternative diagnosis. Timely recognition, multidisciplinary assessment, and histopathological confirmation are essential for establishing the diagnosis and guiding management.

This case also highlights the importance of healthcare-system context when managing rare inflammatory disorders. Differences in access to targeted therapies may influence the practical implementation of international treatment recommendations, reinforcing the importance of individualised and pragmatic care. For clinicians in comparably resourced settings, the practical lesson is to plan early for alternative anti-IL-6 strategies and steroid-sparing regimens in case first-line biologic therapy is inaccessible or not tolerated, so that treatment continuity and clinical outcome do not depend on a single agent being available. Reassuringly, despite the loss of biologic therapy, sustained corticosteroid-sparing immunosuppression allowed meaningful clinical improvement in this patient, with reduced fatigue and weight recovery at follow-up, underscoring that a good outcome remains achievable even when first-line targeted therapy cannot be sustained.
